# Nanoscale Study on Noninvasive Prevention of Dental Erosion of Enamel by Silver Diamine Fluoride

**DOI:** 10.34133/bmr.0103

**Published:** 2024-11-07

**Authors:** Aditi Saha, Yohan Kim, Kack-Kyun Kim, Young J. Kim, Hye Ryung Byon, Seungbum Hong

**Affiliations:** ^1^ Department of Materials Science and Engineering, KAIST, Daejeon 34141, Republic of Korea.; ^2^ Department of Chemistry, KAIST, Daejeon 34141, Republic of Korea.; ^3^ Department of Oral Microbiology and Immunology, Seoul National University, Seoul 03080, Republic of Korea.; ^4^ Department of Pediatric Dentistry, Seoul National University, Seoul 03080, Republic of Korea.

## Abstract

Here, we aimed to demonstrate the efficacy of silver diamine fluoride (SDF) in halting dental erosion caused by dietary selection and offer a potential explanation for the underlying mechanism. We investigated the surface chemical and mechanical characteristics of human tooth enamel when exposed to Coca-Cola from 10 s to 1 h, with and without the topical treatment of SDF. We analyzed the mechanical properties by measuring the enamel surface roughness and elastic modulus using atomic force microscopy and the surface chemical composition through x-ray photoelectron spectroscopy and Fourier transform infrared spectroscopy analyses, with scanning electron microscopy as a supplementary characterization method. After 1 h of immersion in Coca-Cola, the roughness changed from 83 to 287 nm for enamel without SDF treatment and 64 to 70 nm for enamel with SDF treatment. Under the same conditions, the elastic modulus changed from 125 GPa to 13 GPa for enamel without SDF treatment and 215 GPa to 205 GPa for enamel with SDF treatment. Topical coating of SDF onto enamel formed a passivation layer composed of fluorapatite and created added fluorine flux in the system, which protected the teeth from demineralization under Coca-Cola etching, as shown by morphology and chemical composition analysis as well as roughness and modulus characterization. Applying SDF to enamel minimizes changes in chemical compositions and surface roughness while improving enamel elastic modulus.

## Introduction

The prevalence of dental erosion in adults varies widely depending on the population and region. According to a systematic review [1], the prevalence in adults aged 18 to 88 can be between 4% and 88%. In developed countries, the prevalence of dental erosion in permanent teeth ranges from 20% to 45% [2]. This variation is attributed to dietary habits, lifestyle choices, and environmental exposures.

The inherent acid in soft drinks contributes to dental erosion by chemically etching away the dental hard tissue without bacterial involvement. This process is facilitated by the low pH and high buffering capacity of soft drinks, which can significantly lower the surface hardness of enamel and dentine. On the other hand, the sugar in the soft drinks promotes bacterial growth and acid production in the mouth, leading to dental caries [3–6]. Dental erosion and caries from soft drinks are interrelated due to the combined effect of acids and sugars in these beverages.

This erosion can affect primary and permanent dentition. The tooth, the hardest tissue in the human body, is composed of inorganic and organic materials, along with water. Well-structured carbonated hydroxyapatite (HAp) crystals constitute the primary component, comprising 96% of its weight [7]. Tooth enamel, the hardest outer tooth substance, is typically characterized by its robust mechanical attributes throughout an individual’s lifespan. It becomes susceptible to erosion and caries development with consistent intake of sweetened acidic beverages. Carbonated soft drinks react to produce carbonic acid, which erodes tooth enamel [8]. Routine consumption of these beverages is a direct outcome of dietary preference.

As dental erosion can increase the surface roughness of the teeth, it can provide more niches for bacterial adhesion and plaque accumulation, which can lead to caries development [1]. Dental erosion leads to the loss of dental tissue by directly affecting the enamel. Numerous research teams have used diverse biophysical methods, including profilometry and various microscopic techniques such as light microscopy and surface three-dimensional focus variation scanning microscopy, to identify surface roughness and morphology change [9–11]. Although these techniques characterize microscale erosion, erosion at the nanoscale remains relatively less investigated. Atomic force microscopy (AFM) provides high-resolution, subnanometer precision imaging that can detect fine surface alterations in early-stage dental erosion without damaging the fragile enamel layer under erosion unlike surface profilometry, which has lower resolution and can potentially affect the enamel. AFM’s ability to operate in noncontact modes and measure elasticity makes it superior for detailed nondestructive analysis of early dental erosion [12–15].

We attempted to bridge this gap by building on our previous study, which examined the impact of dental erosion on the nanoscale using AFM as a characterization tool [16]. Li et al*.* unveiled alterations in the mechanical and morphological aspects of human tooth enamel surfaces submerged in different sweetened acidic beverages, such as Coca-Cola, Sprite, and orange juice, over varying immersion durations using AFM. The findings revealed enamel softening and roughening as the immersion time in these beverages increased.

Lippert et al. [17] introduced an AFM-based nanoindentation method to investigate dental erosion and explore nanomechanical properties through enamel demineralization and remineralization cycles. Timely dental care is vital because delayed treatment can lead to limited options and eventual tooth loss; hence, early-stage dental intervention is immensely important. Present dental treatments for dental erosion, including fillings, crowns, root canal treatments, and tooth extractions, often involve costly surgical procedures that can induce discomfort (typically requiring local anesthesia) [18–21]. Managing dental issues can incur substantial expenses and significantly affect an individual’s quality of life and self-esteem. Consequently, extensive research has been conducted to uncover the nexus between diet, nutrition, and dental conditions to provide dietary guidelines for prevention. The composition of one’s diet influences not only tooth development but also its subsequent stages [22,23].

Silver diamine fluoride [Ag(NH_3_)_2_F] (SDF) is a localized medicinal agent that is used to decelerate the progression of dental decay in both primary and permanent teeth. This treatment does not cause discomfort, effectively halts dental erosion, and eliminates the need for local anesthesia [24]. SDF functions as a potent inhibitor within affected teeth and impedes dental erosion, particularly in primary teeth and permanent molars [25,26]. Although the exact mechanism by which the silver component of SDF safeguards against dental erosion remains unknown, there is a correlation with the formation of silver phosphate on the enamel surface [27]. However, the staining issue caused by black precipitates at the treatment site must be addressed for SDF to gain acceptance as an effective preventive agent for caries. After SDF treatment, the application of potassium iodide (KI) resolves this problem as the silver iodide precipitate with excess silver is white in color and can be easily washed away with water [28].

Despite some research focusing on the initial stages of erosion, there is a dearth of information on the prevention of dental erosion at the nanoscale level [16]. Additionally, although a substantial amount of work has demonstrated the arrest of dental caries in the dentin region, the early-stage arrest of dental erosion within the enamel region remains underexplored [11]. This study aimed to bridge this gap by offering nanoscale insights to validate the efficacy of SDF + KI application in protecting against dental erosion. Our primary focus is on enamel treated with SDF + KI, intending to showcase the potential for halting dental erosion in its initial phases, that is, in the enamel itself in adults, and propose a plausible mechanism for this process. We used AFM as a foundational characterization tool and the nanoindentation technique to evaluate the elastic modulus and surface roughness alterations on human molar enamel treated with SDF + KI when exposed to an acidic beverage (Coca-Cola). We conducted an in-depth chemical composition analysis using x-ray photoelectron spectroscopy (XPS) to establish the potential mechanisms underlying this process.

## Materials and Methods

### Experimental design

We purchased commercially available soft drinks (Coca-Cola) and SDF & KI kit Riva Star from SDI Limited, Melbourne, Australia. It contains 38% (w/v) diamine silver fluoride and a saturated solution of KI in 2 separate bottles. We treated the enamel sample with a dropwise addition of SDF solution followed by KI solution addition with simultaneous brushing until the white precipitate disappeared from the enamel surface as per the instructions on the Riva Star kit [29]. Subsequently, the sample was dried in air for 1 h and washed with deionized water using a soft brush (Fig. S1).

We classified the enamel samples into 2 groups: group 1, named untreated enamel, which did not go through any treatment before immersion in Coca-Cola, and group 2, called SDF-treated enamel, for the enamel that was treated with SDF + KI solution before immersion in Coca-Cola.

Fluid Cell Lite (Oxford Instruments, Asylum Research) was used to conduct enamel erosion experiments under Coca-Cola. The sample was investigated at room temperature, 25 °C, without any agitation being added while immersed in Coca-Cola in liquid cell. Using a pipette, approximately 2 ml of the soft drink was transferred into the liquid cell to fully immerse the enamel sample. We repeated the immersion procedure over a time span of 2 min with an interval of 10 s to specifically focus on the early stages of erosion, and then increased the interval to 1 min until 10 min of immersion and finally to 1 h of immersion.

After each immersion, the soft drink was pipetted out, and the sample was rinsed with deionized water several times to remove any remaining soft drink. Finally, the samples were dried by using a handheld blower. After the samples were dried, the topography of the enamel surface was characterized by tapping-mode AFM imaging, followed by elastic modulus mapping of the same area.

**Fig. 1.**
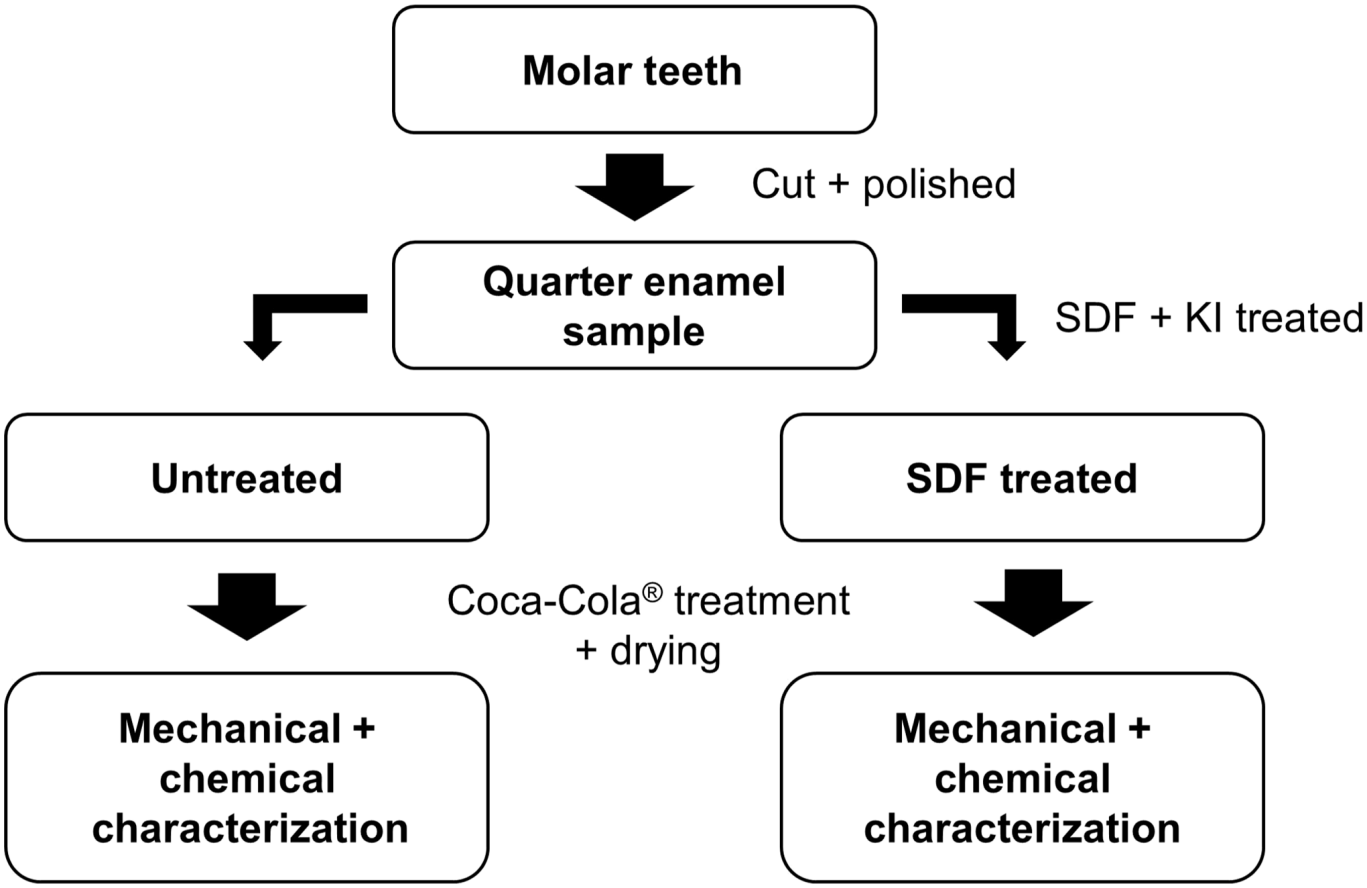
Flowchart of the work.

We investigated the surface chemical composition with XPS and Fourier transform infrared (FTIR) at 0, 2, 10, and 60 min. Additionally, we conducted scanning electron microscopy (SEM) imaging at 0, 2, 10, and 60 min for supporting the AFM topography results. Flow chart of the experimental procedure is shown in Fig. 1.

### Sample preparation

**Fig. 2.**
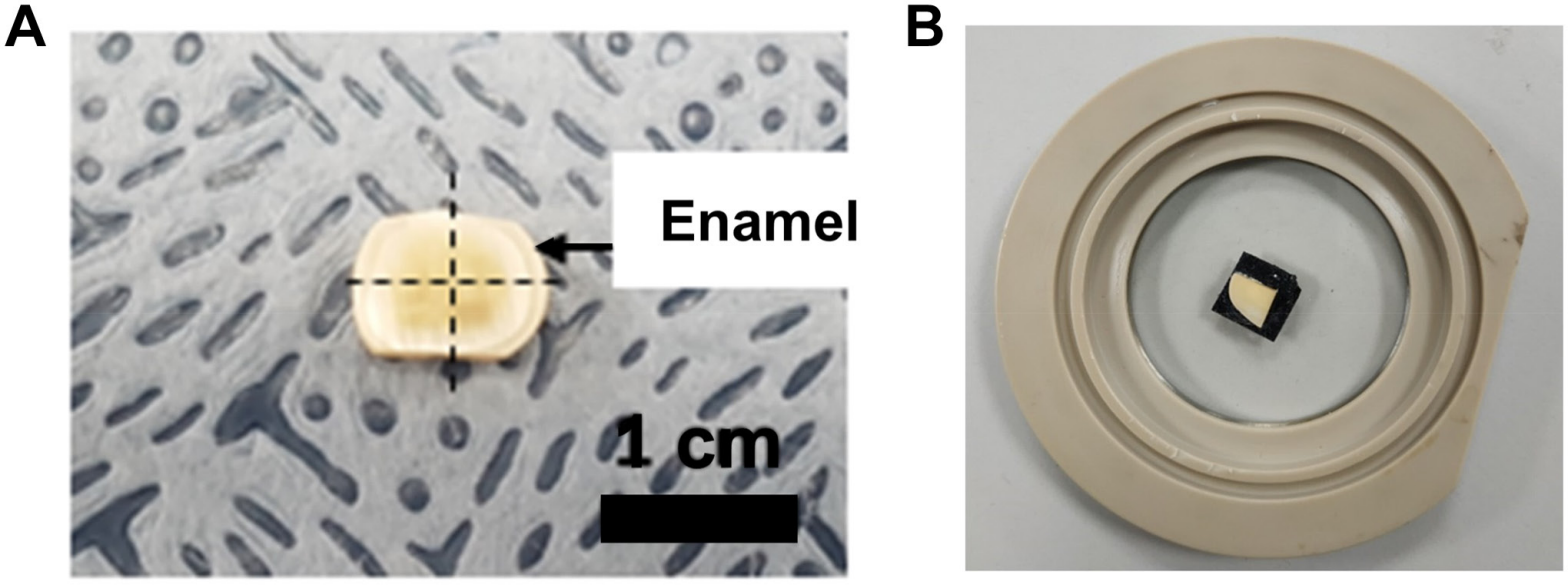
Sample photographs. (A) A 2-mm cross-sectional enamel sample. (B) AFM liquid cell holding the tooth sample.

We collected samples of healthy molars (3rd molar) from volunteers aged 20 to 40 years who visited the KAIST Clinic of Korea Advanced Institute of Science and Technology (KAIST), which was approved by the Institutional Review Board (IRB: KH2020-182) of the KAIST. Informed consent was obtained from all participants, and the relevant guidelines and regulations were followed. The extracted teeth were preserved in Hanks’ balanced salt solution before the experiment [30]. A cross-sectional cut was made to prepare enamel samples from the collected teeth using a diamond saw cutter. Cross-sectional disc-like samples with a thickness of 2 mm were obtained and cut vertically and horizontally to obtain quarter samples. Each quarter-circular sample was used to fit the AFM liquid cell (Fig. 2). We removed any surface contamination by polishing the sample with fine sandpaper (grit 4000) and cleaning the sample with deionized water. Any damage during the sample preparation process was verified using an optical microscope. The sample was then pasted onto the liquid cell using carbon tape, leaving the polished side upward and exposing it to the liquid.

### Roughness and elastic modulus measurements

This study used a commercially available AFM (MFP-3D Origin, Asylum Research, Oxford Instruments, USA) to characterize the surface topography of the enamel sample and the elastic modulus at various immersion times in soft drinks. Point-probe silicon tips (model: DT-NCHR) with diamond coating from NanoWorld with an average spring constant of 80 N/m, elastic modulus (E) of 865 GPa, and an average resonance frequency of 400 kHz were used to perform the imaging and nano-indentation experiments. Every AFM probe must undergo force constant measurement, thermal calibration, and AC tuning to be used in the tapping mode of the AFM. We followed the GetReal calibration procedure provided by the Asylum Research software with the equipment used for the calibration process. The calibration process helps estimate the cantilever sensitivity and spring constant. The calibrated parameters were stored in Igor software with equipment provided by Asylum Research. During calibration, the Igor software automatically generated the force versus indentation curve from the indentation depth and cantilever deflection.

AC tapping mode imaging of AFM (at −10% AC tune frequency) with a scan rate of 0.5 Hz with 512 by 512 pixels was used to measure the surface morphology. The contact force mode of the AFM was used to obtain the force–distance map (F-map) images. The F-map helps estimate the elastic modulus, adhesion, and plasticity of the sample based on the force–distance curve at each point of contact of the probe with the sample in the user’s region of interest. In this experiment, we obtained a matrix of 32 point-by-32 point force–distance curves on an area of 10 μm by 10 μm.

We chose the tapping mode of imaging to observe the surface morphology because it minimizes damage to the surface during imaging. From the surface height image, the Igor software calculates the root mean square roughness (*R*_q_) of the enamel surface. The loading force, which estimates the amount of force applied to the surface when the probe contacts the surface, is maintained at 1.5 μN while obtaining the elastic modulus mapping of the surface.

Upon contact, the cantilever probe detects the mechanical properties, that is, the elastic modulus of the surface. The Hertzian model was used to obtain the elastic modulus mapping. The Igor software was used to investigate the force–displacement curves to obtain the elastic modulus based on the Hertzian model. The point probe has a pyramid shape, and we made a small indentation depth while in contact with the surface; therefore, the spherical type of contact for the Hertzian model can be satisfied. According to the Hertzian model, the elastic modulus can be calculated as follows [31,32].

$$\frac{F}{d^{\frac{3}{2}}}=\frac{4}{3}E_{r}\times R^{\frac{1}{2}}$$ (1)

where *F* is the loading force, *d* is the indentation depth, *E*_r_ is the reduced modulus, and *R* is the tip radius. *E*_r_ defines the elastic deformation that occurs in the sample and probe, which can be further broken down into Eq. 2,

$$\frac{1}{E_{r}}=\frac{1-v_{i}^{2}}{E_{i}}+\frac{1-v_{s}^{2}}{E_{s}}$$ (2)

where *ν*_i_ and *ν*_s_ are the Poisson’s ratios of the indenter and sample, respectively, and *E*_i_ and *E*_s_ are the elastic moduli of the indenter and sample, respectively.

### Enamel surface characterization

We characterized the untreated and SDF-treated enamel surface composition with XPS and FTIR. XPS analysis of the enamel was conducted to analyze the chemical composition of each element using Thermo VG Scientific XPS Nexsa G2 equipment with monochromatic Al Kα as the x-ray source. The XPS analysis was performed at room temperature and 5 × 10^−9^ mbar vacuum with a step size of 0.05 eV over 200-μm area. An ion flood source was used for charge neutralization. Narrow-scan spectra of the C1s region were fitted using the Avantage Data system. The best-fit parameters, peak positions, amplitudes, and full width at half maxima were calculated by a least-squares procedure.

FTIR attenuated total reflection (FTIR-ATR) spectra were obtained in the 400 to 4,000 cm^−1^ range on a Nicolet iS50 with a Germanium window with a resolution of 0.5 cm^−1^ [Thermo Fisher Scientific Instrument, FR-DTGS Detector (12,500 to 350 cm^−1^)].

**Fig. 3.**
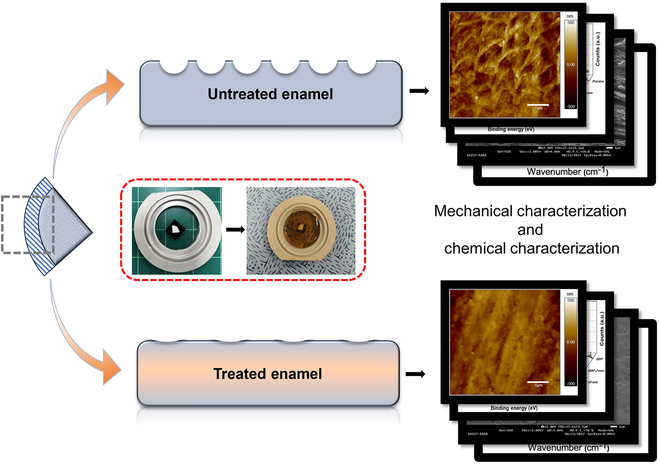
Schematic of the workflow.

SEM images were obtained using JSM-IT800 (JEOL) and FEI Magellan 400. SEM images were taken at 25,000 magnification in the secondary electron detector (SED) mode without any coating on the enamel samples. Schematic of the workflow is given at Fig. 3.

### Statistical analysis

**Fig. 4.**
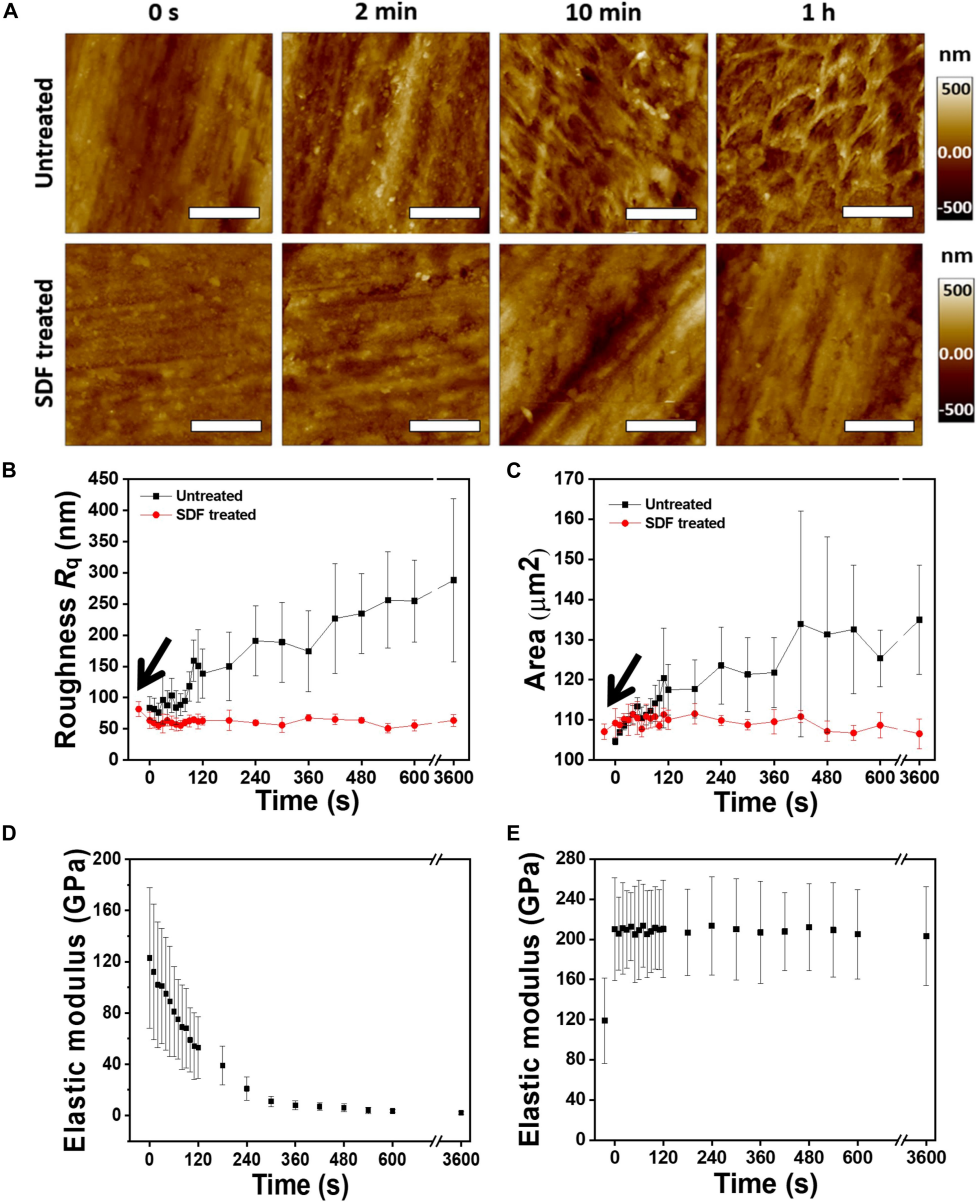
Topography results at 0 s, 120 s (2 min), 600 s (10 min), and 3,600 s (60 min) on Coca-Cola etching. (A) AFM topography images of untreated and SDF-treated enamel with a scale bar of 10 μm. (B) Surface roughness as a function of etching time in Coca-Cola with and without SDF treatment. (C) Surface area as a function of etching time in Coca-Cola with and without SDF treatment. (D) Elastic modulus as a function of etching time for untreated enamel. (E) Elastic modulus as a function of etching time for SDF-treated enamel.

All AFM data were expressed in the form of average ± standard deviation. All statistical analyses were performed with Asylum Research software and Microsoft Excel. Enamel surface *R*_q_ and overall area values were obtained from Asylum Research software (Figs. S2 and S3) after each topography measurement, which were then averaged over 10 randomly selected positions, and the average roughness with the respective standard deviation as a function of the etching time is depicted in Fig. 4B and C. Similarly, we obtained elastic modulus mapping and a matrix of 32 point-by-32 point force–distance curves on an area of 10 μm by 10 μm, and the average elastic modulus value with standard deviation is shown as a function of etching time in Fig. 4D and E.

## Results

### Mechanical characterization of untreated and treated enamel

To investigate the surface morphology and mechanical property changes with and without SDF treatment on enamel surfaces with various etching times in the respective soft drink using AFM, we prepared chronologically etched samples to observe the entire etching procedure. Two categories of samples untreated and SDF treated, which underwent Coca-Cola etching, were analyzed with AFM by observing the topography and elastic modulus changes with increasing etching time.

Figure 4A illustrates the alterations in the enamel surface topography captured through AFM images, showing the impact of varying etching durations for untreated and SDF-treated samples. As the etching duration was extended, keyhole-like micropores were observed on the untreated enamel surface after 2 min, which was absent before any etching. In contrast, no keyhole-like micropores were observed on the SDF-treated enamel surface over the 1-h etching period.

To further understand the etching phenomenon, the correlation between the roughness and surface area with etching time is depicted in Fig. 4B and C, respectively. Notably, upon the application of SDF and KI to the pristine enamel specimen, a considerably reduced or minimal etching impact from the soft beverage was observed. On the polished untreated enamel surface, the initial surface roughness (*R*_q_) of 83 nm increased to approximately 287 nm following a 1-h immersion in Coca-Cola. The SDF-treated enamel surface displayed a considerably smaller alteration in surface roughness when subjected to soft drink etching, remaining within the range of 20 nm throughout the 1-h immersion period. We also found that the enamel surface roughness is reduced upon SDF treatment, as the roughness value reduced from 81 nm to 64 nm. The arrow in Fig. 4B and C shows the roughness and area of the enamel surface prior to application of SDF and KI. Additionally, the surface area with etching time showed a 30.27-μm^2^ change and 3.01-μm^2^ change over the 1-h etching span for the untreated and SDF-treated cases, respectively.

Furthermore, we examined the softening of the enamel surface in the 2 cases by measuring the elastic modulus over the etching span. We found that Coca-Cola etching drastically reduced the elastic modulus of the enamel surface for untreated cases (Fig. 4D) compared with that for SDF-treated cases (Fig. 4E). Our findings revealed a decline in the elastic modulus as the etching time with Coca-Cola increased, plummeting from 125 GPa to 13 GPa within an etching period of 1 h for untreated enamel. In contrast, the reduction in the elastic modulus was minimal to negligible for the SDF-treated enamel samples, shifting modestly from 215 GPa to 205 GPa. It should be noted that treatment with SDF increased the elastic modulus of enamel from 119 GPa to 215 GPa; that is, it increased the mechanical strength of the enamel.

**Fig. 5.**
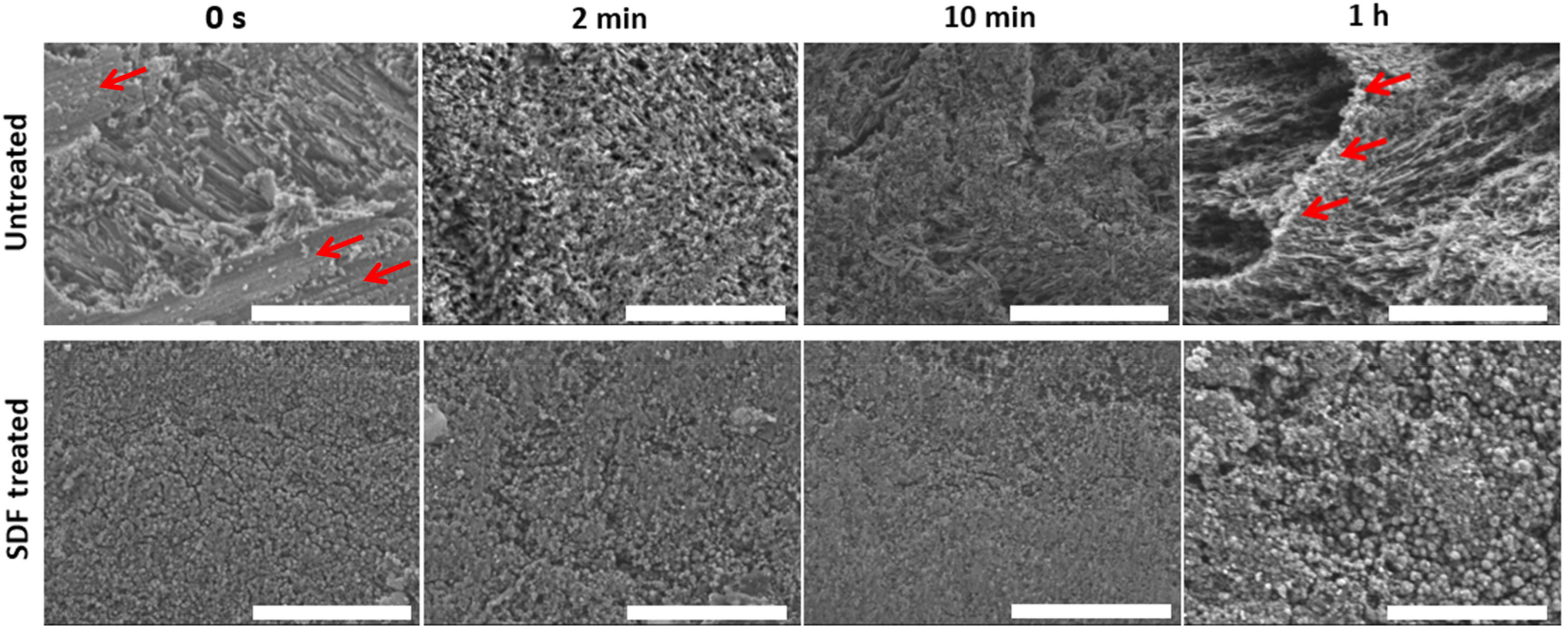
SEM images for untreated and SDF-treated enamel at 0, 2, 10, and 60 min of etching. Scale bars, 1 μm.

Sequential SEM images of the untreated enamel during gradual Coca-Cola etching are presented at a magnification of 25,000, as shown in Fig. 5. The initial cutting patterns stemming from the sawtooth cutter observed for untreated enamel at 0 s (red arrow) were absent after 2 min of etching. Through Coca-Cola etching, the exposure of HAp crystallite structures becomes increasingly prominent, alongside the heightened surface roughness, as corroborated by AFM and SEM images (Figs. 4A and 5). The keyhole type of structure gets prominent after 1 h of immersion in Coca-Cola (Fig. S4). Upon application of SDF and KI onto the enamel, uniform layers of spherical particles over the surface are observed, maintaining their integrity even after an hour of Coca-Cola etching, leading to a modest change in roughness within the range of 10 to 20 nm. These observations were supported by the SEM and AFM images (Figs. 4A and 5).

### Chemical characterization of untreated and treated enamel surfaces

To understand how SDF treatment can help resist such drastic changes in morphology and elastic modulus, we performed chemical characterization of both untreated and SDF-treated enamel surfaces etched for 0, 2, 10, and 60 min using FTIR and XPS.

**Fig. 6.**
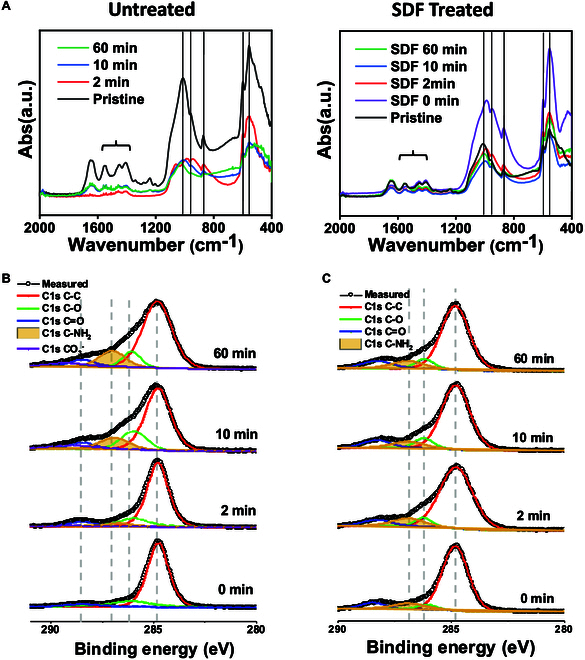
Chemical characterization of untreated and SDF-treated enamel. (A) FTIR spectra enamel as a function of etching time for untreated and SDF-treated enamel. Phosphate 𝜈3 mode is indicated at 1,033 cm^−1^ and carbonate at 859 cm^−1^. (B) C1s XPS spectra of untreated enamel with increased etching time in Coca-Cola. (C) C1s XPS spectra of SDF-treated enamel with increased etching time in Coca-Cola.

In the FTIR spectra of the untreated and SDF-treated enamel samples (Fig. 6A), we could localize the phosphate peaks for ${\mathrm{PO}}_{4}^{3-}$ 𝜈1 mode at 959 cm^−1^, ${\mathrm{PO}}_{4}^{3-}$ 𝜈3 mode at 1,033 cm^−1^, and ${\mathrm{PO}}_{4}^{3-}$ 𝜈4 mode at 562 cm^−1^ with a less intense peak at 603 cm^−1^. These describe the set of characteristic peaks of dental enamel. The type 2 carbonate peak of mode 𝜈2 ${\mathrm{CO}}_{3}^{2-}$ ions substituting ${\mathrm{PO}}_{4}^{3-}$ sites is also around 872 cm^−1^. The type 2 carbonate peak of mode 𝜈 is present at 1,415 and 1,457 cm^−1^. Type 1 carbonate ${\mathrm{CO}}_{3}^{2-}$ substituting OH^−^ in 𝜈3 mode is located around 1,545 cm^−1^. With progressive etching, the phosphate peak intensity decreases to half by 2 min, followed by a decrease of 80% by the end of 1 h without any change in the peak position. However, once SDF is applied to the treated enamel, there is no shift in the phosphate peaks in the respective peak position with no intensity change for up to an hour of etching [33,34]. It should be noted that the shift in the peak after the application of SDF and KI may be due to environmental change, primarily due to the presence of fluorine.

Figure 6B and C shows the C1s XPS spectra of the untreated and SDF-treated enamel, respectively. The C1s spectra can be deconvoluted into 5 peaks corresponding to (C-C, C-O, C=O, C-NH_2_, and CO_3_^−^). The hydrocarbon peak is shown in red, the C-O peak is shown in green, and the C=O peak is shown in blue. The relative area changes related to the possible reaction products are presented in Table 1 and shown in Fig. S5. As the immersion time in Coca-Cola increased, the hydrocarbon peak intensity decreased, and simultaneously, C-O, C=O, and C-NH_2_ increased, confirming that the enamel was demineralized, and its amino acid structure was exposed [35,36]. SDF-treated enamel showed a higher resistance toward demineralization than untreated enamel, as confirmed by the percentage area change of the deconvoluted peaks.

**Table 1.** Area percentage change for deconvoluted C1s XPS spectra into C-C, C-O, C=O, and C-NH_2_ bonds as a function of etching time for untreated and SDF-treated cases

Group	Etching time (min)	Deconvoluted area (%)				
		C1s C-C	C1s C-O	C1s C=O	C1s C-NH_2_	C1s CO_3_^−^
Untreated	0	78.38	16.56	5.05	0	-
	2	71.10	16.61	6.27	5.00	0.01
	10	59.44	17.22	6.06	11.75	5.50
	60	57.64	12.13	8.51	17.74	3.98
SDF treated	0	80.06	2.01	7.33	8.62	-
	2	81.11	4.87	5.65	8.35	-
	10	75.21	7.61	8.50	8.56	-
	60	74.32	7.28	8.70	9.68	-

O1s XPS spectra of the untreated and treated enamel are shown in Fig. S6. The O1s spectra can be deconvoluted into 6 peaks resulting from the O1s peak from oxygen bonded with phosphorus (shown in red) and the O1s peak from oxygen bonded with calcium (shown in green) in HAp. The C=O–carbon-oxygen carboxylic acid peak is shown in blue. We observed that for the untreated enamel, the O-P peak simultaneously decreased the C-O, C=O, and Ca-OH peaks, supporting the demineralization of the enamel [37,38]. After treatment with SDF, the respective relative area changes of the convoluted O1s peaks varied significantly less than that of the untreated enamel (Table 2 and Fig. S7).

**Table 2.** Area percentage change for deconvoluted O1s XPS spectra into O-P of HAp, O-Ca of HAp, C-O, C=O, and CO^−^_3_ bonds as a function of etching time for untreated and SDF-treated cases

Group	Etching time (min)	Deconvoluted area (%)				
		O-P of HAP	O-Ca of HAP	C-O	C=O	CO^−^_3_
Untreated	0	85.81	5.16	1.95	-	-
	2	80.06	11.29	6.52	2.13	0.19
	10	58.47	19.02	8.54	13.97	0.23
	60	53.36	18.03	9.21	19.40	0.28
SDF treated	0	81.82	3.38	12.50	2.30	-
	2	74.04	3.19	19.83	2.94	-
	10	71.79	2.51	20.07	5.63	-
	60	71.51	2.07	21.22	5.11	0.09

Figure S8 shows the P2p XPS spectra of the untreated and treated enamel. The P2p spectra show a split of P2p_3/2_ and P2p_1/2_ for spin-orbit coupling in the case of phosphorus [37,39]. In this study, we observe a 0.6-eV shift toward higher energy with increasing etching time in Coca-Cola for untreated and a 0.15-eV shift toward higher energy for the SDF-treated sample. With increasing etching time for the untreated enamel, more phosphate is produced as the enamel undergoes demineralization, resulting in a shift in the phosphate peak to a higher energy. In addition, the relative area change with progressive etching time was smaller for the treated enamel (Table S1 and Fig. S9).

**Fig. 7.**
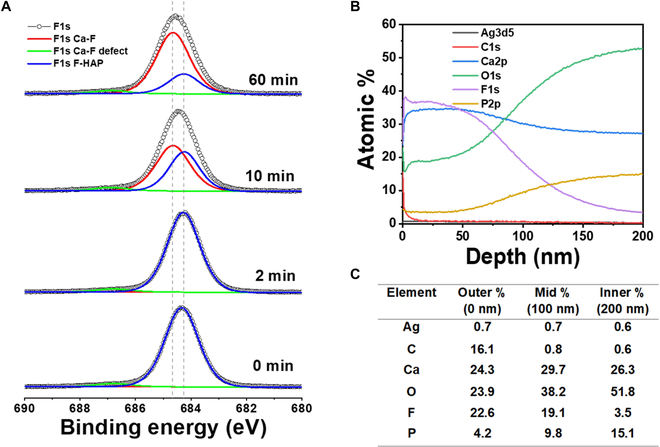
Presence of fluorine in SDF-treated enamel. (A) F1s XPS spectra of SDF-treated enamel. (B) Depth profile of SDF-treated enamel. (C) Atomic percentage of the elements over the depth profile range.

The chemical state of silver was analyzed using XPS, revealing distinct Ag3d peaks in the groups treated with SDF. Two specific peaks at binding energies of 374.1 and 368.1 eV corresponding to the Ag3d_5/2_ and Ag3d_3/2_ electrons of metallic silver [40] are observed (Fig. S10). The spin energy separation of 6.0 eV confirmed the presence of metallic silver on the SDF-treated enamel surface. Also, with an hour of immersion time in Coca-Cola, a shift of 0.15 eV toward lower energy is observed, which could result from Ag^+^ state of silver [41]. The chemical state of Ca was also analyzed with XPS (Fig. S11), which revealed distinct peaks of 347 and 350.6 eV corresponding to Ca2p_3/2_ and Ca2p_1/2_, respectively [42]. With increasing immersion time in Coca-Cola, we observe a shift of 0.5 eV over an hour of immersion, indicating the presence of CaF_2_ [43]. Additionally, the presence of fluorine was detected upon SDF treatment as shown in Fig. 7A. Fluorine F1s peak corresponding to fluorapatite was observed at 684.6 eV [38]. With increasing immersion time, the F1s peak corresponding to CaF_2_ at 684.9 eV became prominent [44]. Furthermore, we performed depth profile over 200 nm for SDF-treated enamel, which revealed a near-surface fluorine content of 38.2% (by atomic weight), which gradually decreased by half at a depth of 100 nm (Fig. 7B). Over the 200-nm depth, silver content was found to decrease from 0.7% to 0.6%. Additionally, phosphorus and oxygen presence was found to increase from 4.2% to 15.1% and 23.9% to 51.8%, respectively (Fig. 7C).

## Discussion

Based on the chemical analysis, possible reactions of enamel with SDF are as follows:

Iso-ionic exchange of F^−^ for OH^−^ in apatite [45,46]:

$${\mathrm{Ca}}_{10}{\left({\mathrm{PO}}_{4}\right)}_{6}{\left(\mathrm{OH}\right)}_{2}+2F^{-}\to {\mathrm{Ca}}_{10}{\left({\mathrm{PO}}_{4}\right)}_{6}F_{2}+2{\mathrm{OH}}^{-}$$ (3)

Apatite dissolution with SDF into CaF_2_ and silver phosphate formation:

$$\begin{array}{c} {\mathrm{Ca}}_{10}{\left({\mathrm{PO}}_{4}\right)}_{6}{\left(\mathrm{OH}\right)}_{2}+20\mathrm{Ag}{\left(NH_{3}\right)}_{2}F+40H_{2}O\to 10{\mathrm{CaF}}_{2}\\ +6{\mathrm{Ag}}_{3}{\mathrm{PO}}_{4}+40{\mathrm{NH}}_{4}\mathrm{OH}+2{\mathrm{Ag}}^{+}+2{\mathrm{OH}}^{-} \end{array}$$ (4)

Excess silver, which is reported to darken teeth, reacts with KI to form a yellowish precipitate of silver iodide, which is then washed away with deionized water.

Coca-Cola, which contains phosphoric acid, exhibits a pH of 2.6 [47]. Beverages with a pH lower than 4 are responsible for irreversible dental erosion. Beverages that we usually consume are classified based on their pH level as extremely erosive (pH < 3.0), erosive (3.0 ≤ pH ≤ 3.99), and minimally erosive (4.0 ≤ pH < 5.5) [47]. Beverage-induced dental erosion primarily results from phosphoric acid and/or citric acid, which are triprotic acids containing 3 available [H^+^] ions that facilitate proton-induced dissolution [48,49]. The introduction of SDF strengthens the enamel by substituting hydroxyl ions with fluoride ions within the HAp structure, thus modifying the critical pH range. Moreover, the application of SDF and KI creates an additional fluoride reservoir.

**Fig. 8.**
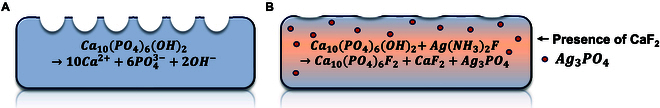
Schematic of demineralized enamel. (A) Untreated enamel. (B) SDF-treated enamel.

As there is an increasing presence of CaF_2_ (Fig. 7A and Fig. S9) and a presence of Ag^+^ (Fig. S10) with increasing etching time in Coca-Cola, the iso-ionic exchange of F^−^ for OH^−^ in apatite and apatite dissolution procedures falls in line with the possible mechanism. It indicates the presence of a fluorine reservoir stemming from the effects of SDF treatment. Furthermore, the presence of silver remained consistent at 0.7 to 0.6% at a depth of 200 nm, implying a distribution of silver permeating the surface. It might be argued that the prevention effect of SDF is originated from the veneering effect. However, in the case of SDF application followed by KI, there should be less tendency of any precipitate to stick on the surface. While SDF application followed by KI strengthens enamel and provides some surface-level protection, it does not alter the appearance of the teeth in a significant or permanent way, nor does it cover imperfections like a veneer would. To double check this argument, we conducted a positive control experiment with a manicured enamel surface and used the normalized roughness and elastic modulus as a deciding factor (Fig. S12). The manicured surface showed a significant change in both roughness and elastic modulus compared to SDF-treated case, confirming our hypothesis. Based on this reasoning and our chemical and mechanical property analyses of the untreated and SDF-treated enamel surfaces, we established a model to understand how SDF treatment withstood the enamel-etching capabilities of Coca-Cola (Fig. 8). Additionally, the presence of Ag_3_PO_4_ acts as a phosphate reservoir, and the effectiveness of silver as an antibacterial agent is well established in dentistry [27].

In conclusion, we demonstrated the efficacy of SDF in resisting dental erosion caused by the consumption of soft beverages. AFM was used to investigate the surface roughness and elastic modulus of the enamel in the untreated and SDF-treated cases under soft drink etching. In addition, XPS, FTIR, and SEM were used to explain the chemical changes during the process. After immersion in Coca-Cola for 1 h, we found that the surface roughness of untreated enamel increased from 83 to 287 nm, while SDF-treated enamel showed a change from 64 nm to 70 nm. Under the same conditions, the elastic modulus decreased from 125 GPa to 13 GPa for the untreated enamel and 215 GPa to 205 GPa for the SDF-treated enamel. Applying SDF to the enamel resulted in the iso-ionic exchange of hydroxyl ions with fluorine ions. This resulted in a protective layer comprising fluorapatite and generated an additional fluorine flux in CaF_2_. It enhances the mechanical properties of the enamel by replacing surface HAp with fluorapatite and creating an additional fluorine flux. Our findings provide evidence that in the field of family dentistry, SDF treatment can be effective on sound teeth as a preventive measure against the formation of dental erosion leading to caries from dietary choices and can be introduced as an alternative painless and affordable early-state measure in place of later-stage painful and expensive traditional surgical treatments.

## Data Availability

The data are freely available upon request.
