# Supplementary material for: Nanoscale Study on Noninvasive Prevention of Dental Erosion of Enamel by Silver Diamine Fluoride
Source: Biomater Res. 2024 Nov 7;28:0103. doi: 10.34133/bmr.0103 (PMC11558617; doi:10.34133/bmr.0103)
Supplement: Supplementary 1 — Figs. S1 to S12 Table S1 [file bmr.0103.f1.docx]

Supplementary materials

Title

Nanoscale study on noninvasive prevention of dental erosion of enamel by silver diamine fluoride

Short title: Enamel protection by SDF

**Authors**

Aditi Saha^1^, Yohan Kim^2^, Kack-Kyun Kim^3^, Young J. Kim^4^, Hye Ryung Byon^2^,

Seungbum Hong^1*^

**Affiliations**

^1^Department of Materials Science and Engineering, KAIST, Daejeon, 34141, Republic of Korea.

^2^Department of Chemistry, KAIST, Daejeon, 34141, Republic of Korea.

^3^Department of Oral Microbiology and Immunology, Seoul National University, Seoul, 03080, Republic of Korea.
^4^Department of Pediatric Dentistry, Seoul National University, Seoul, 03080, Republic of Korea.

^*^Address correspondence to: [seungbum@kaist.ac.kr](mailto:seungbum@kaist.ac.kr)

Sample preparation


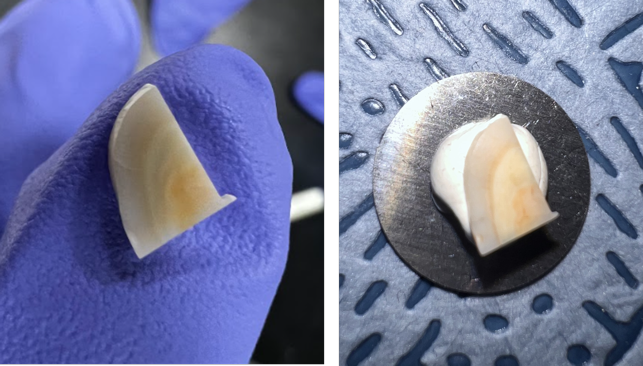


**Fig. S1.** Teeth sample before (left) and after (right) SDF+KI application.

Statistical Analysis

Asylum research software provides tools for appropriate statistical analysis. For example, the analyze tab shown in Figure S2 provides the statistics on images such as R_q_ and R_a_. R_q_ is defined as the standard deviation of the calculated roughness, and calculated over the whole image when unmasked. The SW was used to analyze the topography images and F-map (Section 7.3, The Analyze panel of AR SPM Software Guide_19C, <https://support.asylumresearch.com/articles/documentation/manuals>). The software uses the equation,

$\sqrt{\frac{1}{n-1}\sum{(x}_{i}-\bar{x})^{2}}$ (1)

to calculate the R_q_ values corresponding to the whole image area.


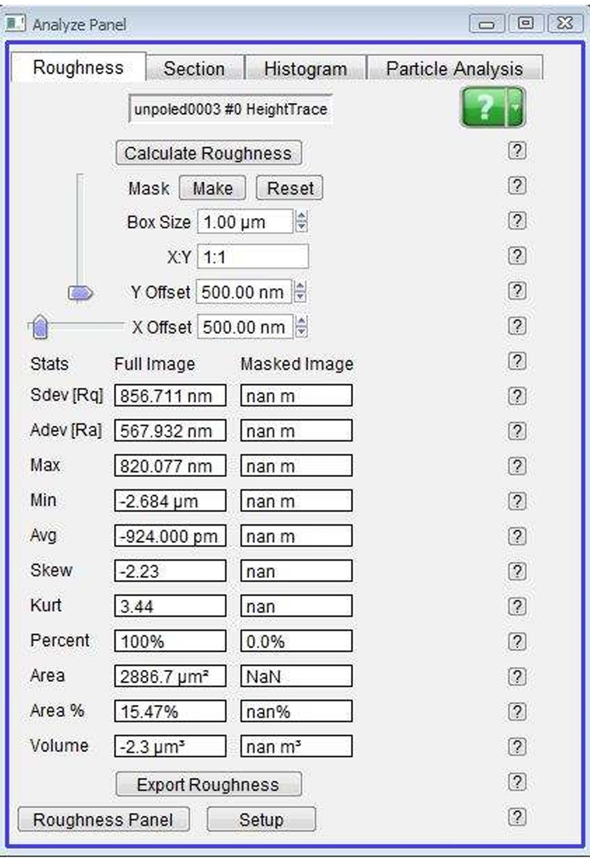


**Fig. S2.** Analyze Panel.

For the topography, randomly selected 10 different positions are considered for the 10 μm by 10 μm area and the average roughness is plotted.


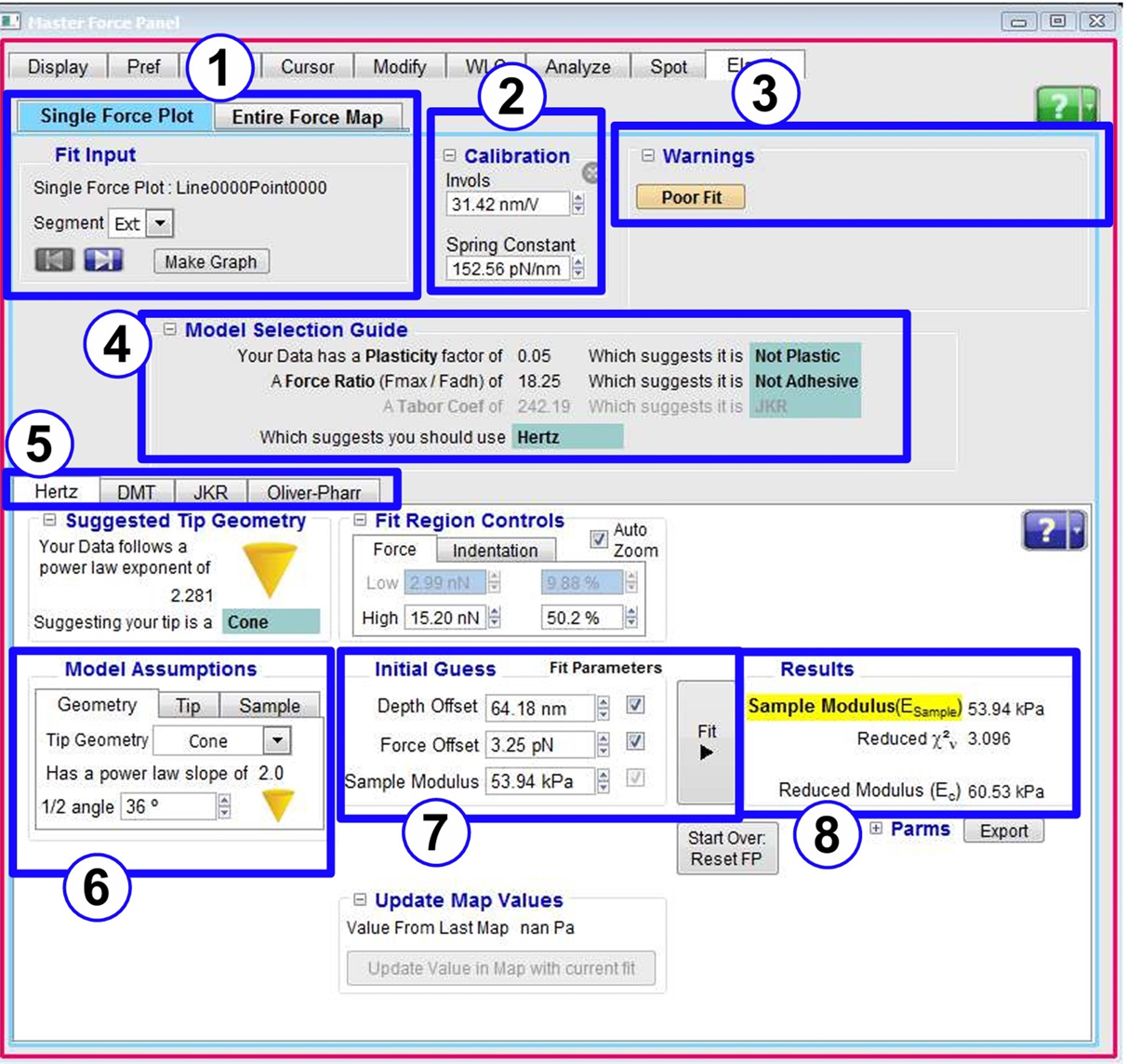


**Fig. S3**. Elastic Tab.

Additionally, as described in the experimental design section, we performed a 32 by 32 grid Fmap over the 10 μm by 10 μm area, which is averaged by the software with the standard deviation. The force tab of the software (Fig. S3) is used to obtain the fitted force map, where Hertzian model is used. Hence, the software is capable of statistical analysis.

Results


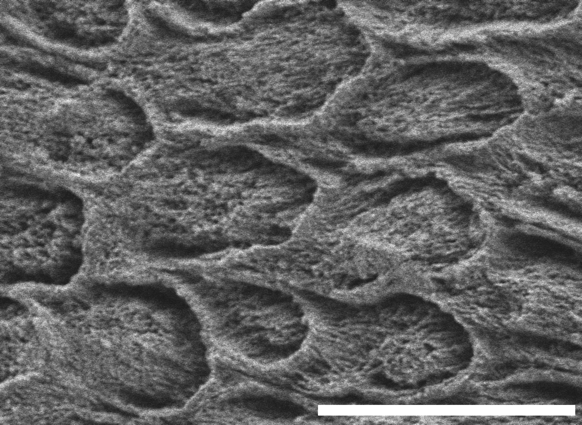


**Fig. S4.** SEM image of untreated enamel at 60 minutes of erosion, with scale bar of 10 μm.

**
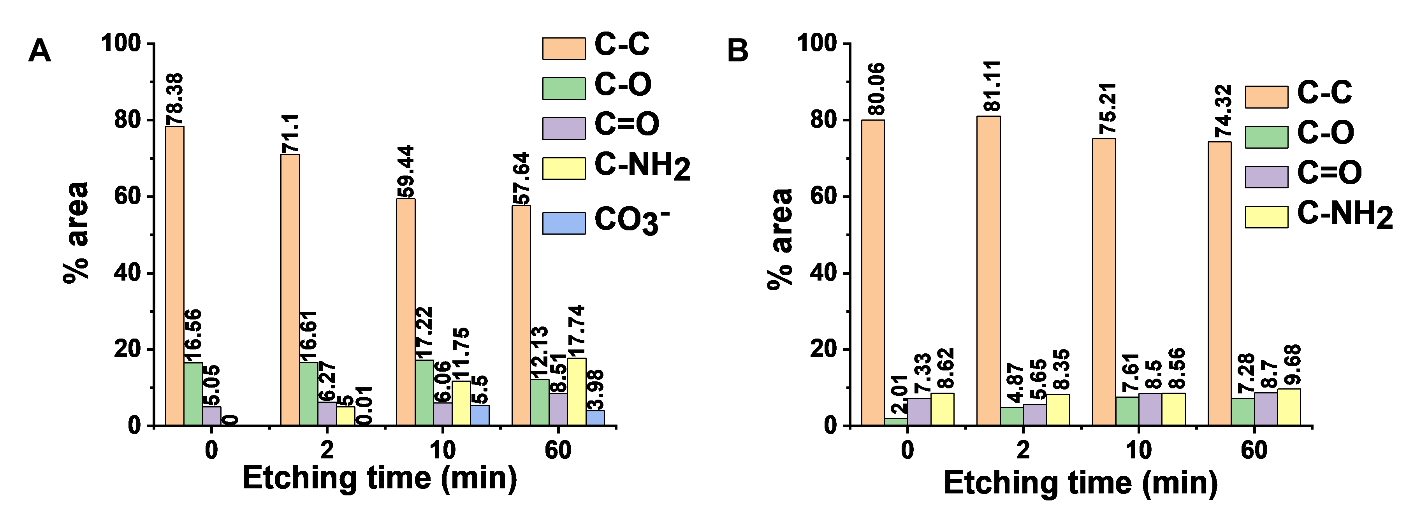
**

**Fig. S5.** Area percentage change for deconvoluted C1s XPS spectra into C-C, C-O, C=O, and C-NH_2_ bonds as a function of etching time. (A) untreated and (B) SDF-treated cases.

**
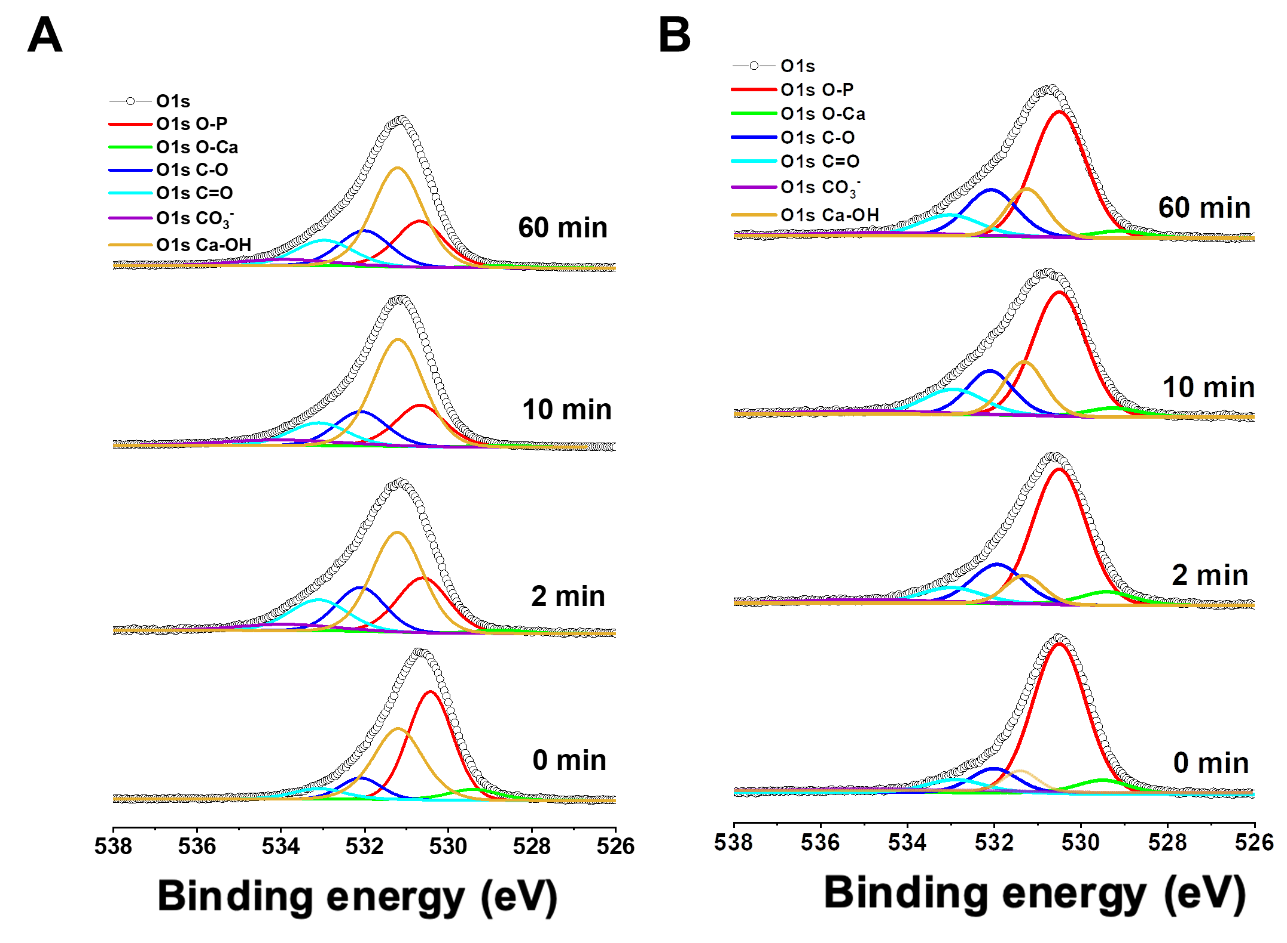
**

**Fig. S6.** O1s XPS spectra with increased etching time by Coca-Cola^®^. (A) untreated, and (B) SDF-treated.


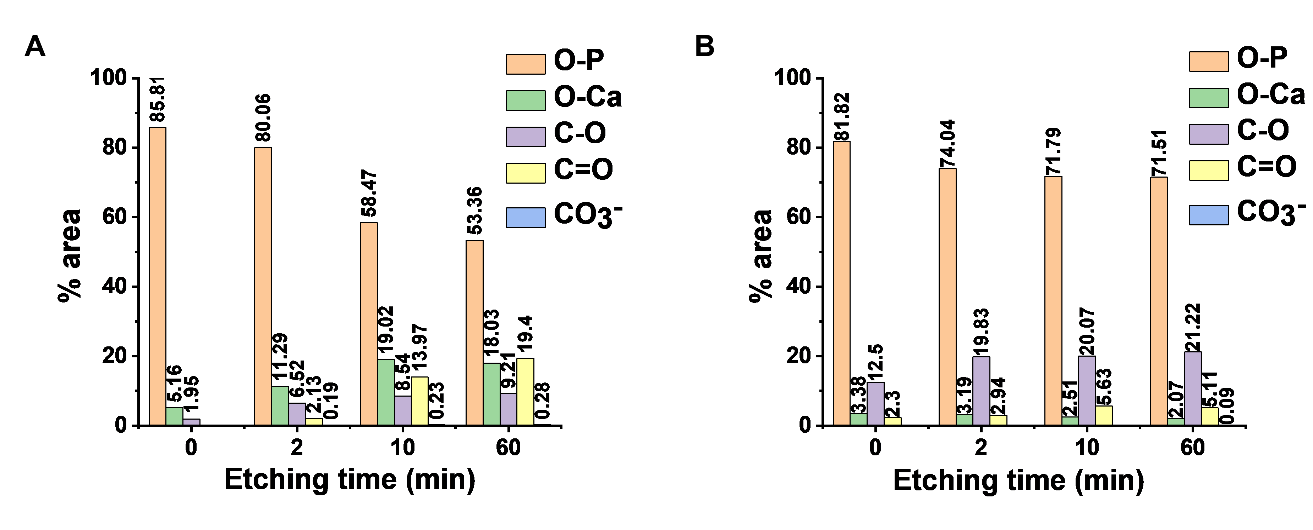


**Fig. S7.** Area percentage change for deconvoluted O1s XPS spectra into O-P of HAP, O-Ca of HAP, C-O, C=O, and CO^-^_3_ bonds as a function of etching time. (A) untreated and (B) SDF-treated cases.

**
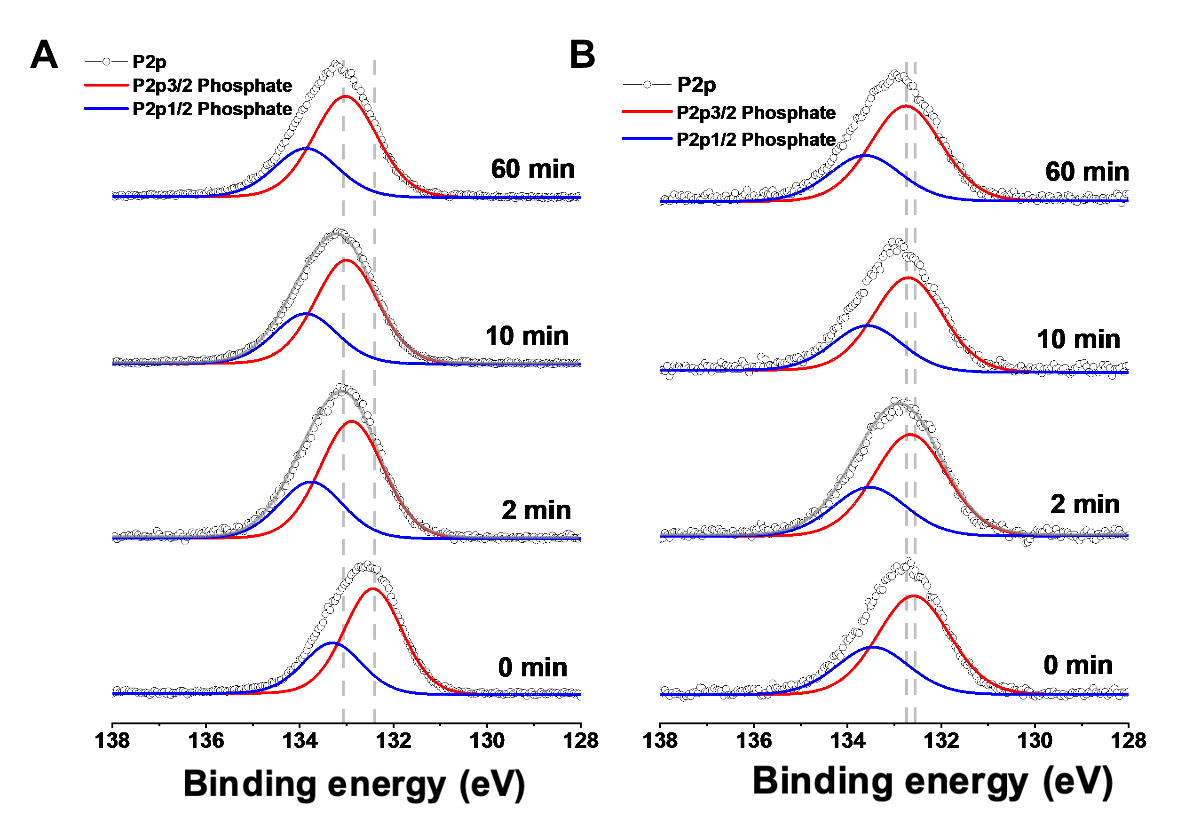
**

**Fig. S8.** P2p XPS spectra with increased etching time in Coca-Cola^®^. (A) untreated, and (B) SDF-treated.


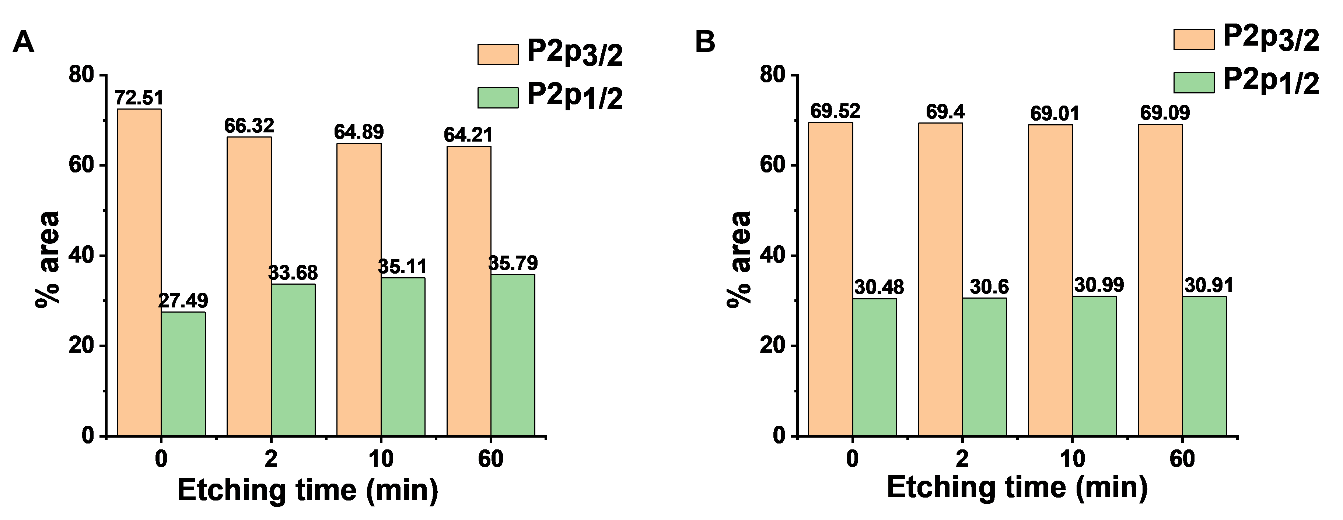


**Fig. S9.** Area percentage change for deconvoluted P2p XPS spectra into P2p_3/2_ and P2p_1/2_ bonds as a function of etching time. (A) untreated and (B) SDF-treated cases.


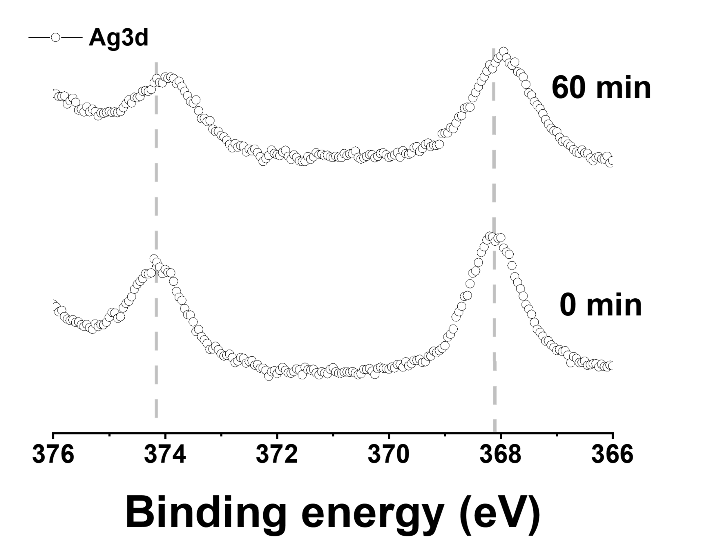


**Fig. S10.** Ag3d XPS spectra of SDF-treated enamel after 0- and 60-min etching time in Coca-Cola^®^.


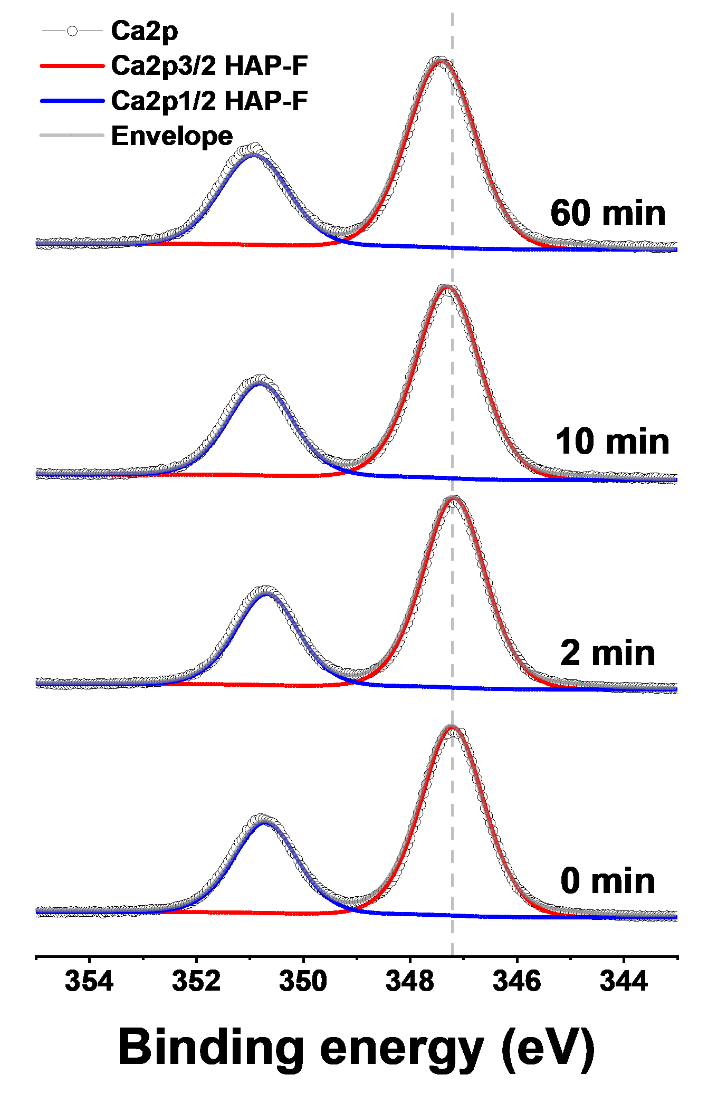


**Fig. S11.** Ca2p XPS spectra of SDF-treated enamel with increased etching time in Coca-Cola^®^.


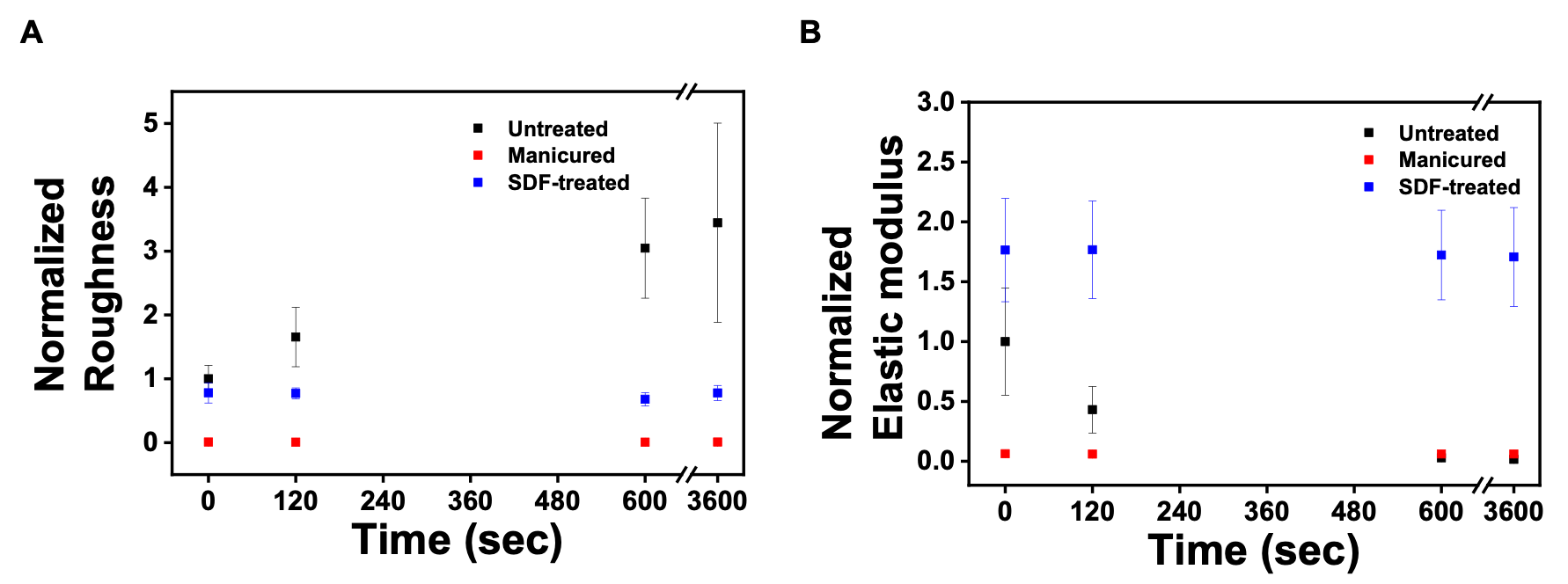


**Fig. S12.** Topography results at 0, 120 s (2 min), 600 s (10 min), and 3600 s (60 min) by Coca-Cola^®^ etching. (A) Normalized roughness changes for untreated, manicured and SDF-treated enamel surface. (B) Normalized elastic modulus changes for untreated, manicured and SDF-treated enamel surface.

**Table S1.** Area percentage change for deconvoluted P2p XPS spectra into P2p_3/2_ and P2p_1/2_ bonds as a function of etching time for untreated and SDF-treated cases.

| Group | Etching time (min) | Deconvoluted area (%) | |
| --- | --- | --- | --- |
|  |  | **P2p_3/2_** | **P2p_1/2_** |
| **Untreated** | 0 | 72.51 | 27.49 |
|  | 2 | 66.32 | 33.68 |
|  | 10 | 64.89 | 35.11 |
|  | 60 | 64.21 | 35.79 |
| **SDF-treated** | 0 | 69.52 | 30.48 |
|  | 2 | 69.40 | 30.60 |
|  | 10 | 69.01 | 30.99 |
|  | 60 | 69.09 | 30.91 |
